# Review of Smart Services for Tinnitus Self-Help, Diagnostics and Treatments

**DOI:** 10.3389/fnins.2018.00541

**Published:** 2018-08-20

**Authors:** Sven Kalle, Winfried Schlee, Rüdiger C. Pryss, Thomas Probst, Manfred Reichert, Berthold Langguth, Myra Spiliopoulou

**Affiliations:** ^1^Faculty of Computer Science, Otto-von-Guericke-University Magdeburg, Magdeburg, Germany; ^2^Department of Psychiatry and Psychotherapy of Regensburg University, Regensburg, Germany; ^3^Institute of Databases and Information Systems, Ulm University, Ulm, Germany; ^4^Department for Psychotherapy and Biopsychosocial Health, Danube University Krems, Krems an der Donau, Austria

**Keywords:** tinnitus treatment, tinnitus monitoring, smart technologies, internet-based treatments, iCBT, tinnitus masking, tinnitus, mobile crowd sensing

## Abstract

In the recent years, there has been an increasing interest in the potential of internet- and smartphone-based technologies for the support of tinnitus patients. A broad spectrum of relevant approaches, some in the form of studies, others in the form of market products, have been mentioned in literature. They include auditory treatments, internet-based Cognitive Behavioral Therapy (iCBT), serious games, and questionnaires for tinnitus monitoring. The goal of this study is to highlight the role of existing internet-based and smart technologies for the advancement of tinnitus clinical practice: we consider contributions that refer to treatments and diagnostics, and we include contributions refering to self-help measures. We elaborate on the potential and challenges of such solutions and identify constraints associated to their deployment, such as the demand for familiarity with internet-based services and the need to re-design interactive services so that they fit on the small surface of a smartwatch.

## 1. Introduction

Tinnitus is defined as the perception of a sound with a lack of an evident external stimulus to that sound. About 10–15% of the general population is affected by tinnitus, whereas for 1–2% the tinnitus is so severe that it directly affects their quality of life, according to Baguley et al. (2013). Modern technologies, including internet-based services and smart devices, are in principle appropriate for reaching and assisting those patients.

In this work, we survey internet- and smartphone-based solutions for tinnitus treatment as well as for everyday monitoring of tinnitus severity. Two earlier reviews have addressed the role of such technologies for tinnitus therapy, namely the works of Nyenhuis et al. (2013) and Greenwell et al. (2016a), which investigate the efficacy of self-help interventions. Since the technologies of relevance in the context of our study lend themselves to self-help measures, there is an overlap between our materials and theirs. As opposed to these reviews however, our aim is not to discern the efficacy of such solutions, but rather to assess the coverage of this technology in scientific literature with respect to treatments, diagnostics and self-help. The secondary aim reported by Greenwell et al. (2016a), namely “identifying what intervention techniques are used within the interventions” (page 83), relates more to our primary goal. However, our emphasis is not on “identify[ing] and describ[ing] the ‘active ingredients’ of the interventions” (page 83), but rather on identifying the types of treatment and the types of self-help being offered, the particular challenges that have been addressed in order to make such services as accessible and useful as possible, and the technical limitations that have not been solved yet.

The recent review of Lui et al. (2017) shows some similarities to our approach as well. Lui et al. (2017) study mobile applications for mental health, concentrating on evidence-based apps in the psychotherapeutic context and investigating their efficacy. Tinnitus is not among the conditions they investigate, albeit they discuss apps that support cognitive behavioral therapy (CBT), which belongs to the widespread tinnitus treatments. Accordingly, they do not address the issue of effectiveness of such treatments with respect to tinnitus nor the design of studies involving the use of smartphones and similar technologies among tinnitus patients, as we do here.

## 2. Review design

### 2.1. Specific aims of the review

We investigate how internet-based and smart services are taken up clinically for tinnitus diagnostics and treatments, including self-help measures. We aim to identify key findings, key contributors and key challenges.

We solely include scientific articles that report on treatments, diagnostics and self-help for tinnitus, but no gray literature. In addition to the main review, we performed a market overview, on which we report in section 2.3.5. Note that in this market overview it has not been possible to distinguish between services intended exclusively for treatment and those concerning well-being in general.

Internet- or smartphone based treatments for tinnitus can be mainly differentiated into auditory and psychological treatments. *Auditory treatments* encompass (i) environmental sound generators, (ii) tinnitus maskers that generate low-level broad-band noise, (iii) hearing aids, and (iv) devices that combine some of the above, all aiming to reduce or mask the tinnitus percept. *Psychological treatments/interventions*, especially Cognitive Behavioral Therapy (CBT), are also investigated in our search, because earlier studies (e.g., by Martinez-Devesa et al., 2010; Cima et al., 2012) have shown significant effects of CBT on the quality of life and on the depression scores of tinnitus patients. Medications are not considered, since we expect that internet-based and smartphone technologies are used for tinnitus medications in the same way as for medications of other chronical diseases (like diabetes or hypertension), namely to acquire information about the medication, as well as its effects and side-effects, to set reminders about taking the medication and to monitor live signals.

*Internet- and smartphone based Diagnostics* encompass the use of questionnaires to collect patient assessments and further information, as well as crowdsensing with the help of hardware, e.g., for tinnitus matching. It should be stressed that diagnostics are often linked to treatments. Finally, *Self-help* refers to both helping the patients to cope with tinnitus as well as helping the patients in diagnostics, e.g., by tinnitus matching.

### 2.2. Approach for the collection and selection of contributions

Our search approach encompasses a broad keyword-based search in Google Scholar using the top-20 results for each keyword, the identification of key authors, a targeted search in selected journals using keywords and key authors, a second search in Google Scholar following the same method as the first one, to update our document collections with more recent publications, and a search for mobile apps released for different smart phones.

#### 2.2.1. Broad keyword-based search in two runs

We used Google Scholar to collect articles on the following keywords: “tinnitus smart,” “tinnitus smart phone,” “tinnitus smartphone,” “tinnitus internet based,” “tinnitus internet based cbt,” “tinnitus icbt,” “tinnitus sound therapy smart,” “tinnitus mobile,” “tinnitus online based,” and “tinnitus internet acceptance” (excluding citations and patents). We performed one run on August 1, 2016, and a complementary one on June 11, 2017 to add more recent publications.

To decide which articles we should finally include, we first inspected title and abstract. During this inspection, we excluded articles that had reported on how the internet was used to recruit study participants as well as articles investigating the impact of internet usage (e.g., excessive usage) on the well being of patients. In some cases, it has become necessary to also inspect the contents of the articles themselves.

In the first run, out of the 45 articles that potentially seemed to be relevant, actually, 19 were considered as relevant and were thus included in our study.

The second run delivered 27 new articles that were possibly relevant to this work. Of these 27 articles, 11 were indeed relevant to this review. Moreover, there was another study not being traced by the described search procedure, but being added to the present review as the authors consider it as essential (Henry et al., 2012).

#### 2.2.2. Targeted search in Scopus and in PubMed using keywords

As next step, we performed a more targeted journal search in PubMed, focussing on medical advances, and in Scopus. The search on these platforms was performed on February 27, 2017. For both collections we used the same keywords as before, and we acquired 18, respectively 19 papers we marked as potentially relevant. After inspection and duplicate removal, 26 papers remained, of which 11 were considered to be relevant.

#### 2.2.3. Search for relevant mobile apps

We performed a manual search through the most popular stores for smartphone applications. Apps were searched in the browser version of Google's PlayStore, Apple's AppStore by using their software iTunes, and Microsoft's store included in the Windows 10 Mobile OS, using the keyword “tinnitus.” The search run was performed on February 28, 2017. We then inspected the textual descriptions of the apps and removed those not related to tinnitus, e.g., wellness apps, as well as packages composed of multiple apps for different purposes. The results are discussed in section 2.3.5.

### 2.3. Overview of the collected contributions

**Figure 1:**
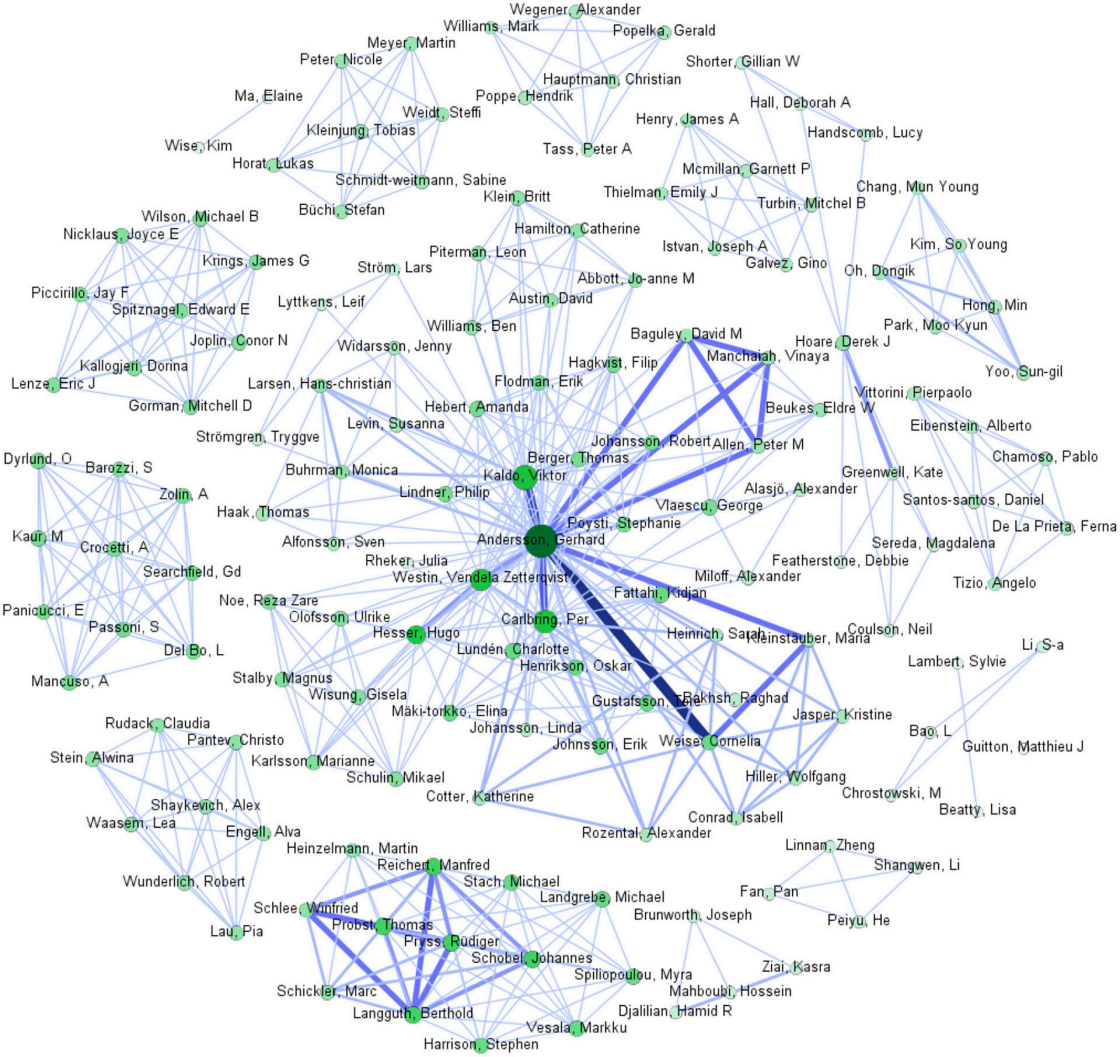
Co-Authorship network: (i) Node size and node color intensity reflect the number of articles that this author wrote and were found relevant for our review; the larger and darker, the higher the number (e.g., Gerhard Andersson has the most works with 21, next is Viktor Kaldo with 7) (ii) Edge thickness and edge color intensity reflect the number of articles co-authored by the nodes linked through the edge; the thicker and darker, the higher the number (e.g., the thickest edge connects Gerhard Andersson and Cornelia Weise, who have co-authored 7 articles). We used the Sci2 Tool [https://sci2.cns.iu.edu/] to extract the network from the bibtex file containing all papers that were identified as relevant. After extracting the network, we used Gephi [https://gephi.org/] (which can be directly called through the Sci2 Tool), to draw the graph. After picking parameters for the colors and size of nodes and edges, we used the layout algorithm “Fruchterman Reingold,” which is implemented in Gephi, for achieving a more visually appealing graph-layout.

We have organized the studies we found in the five areas described hereafter. Further, we have used the article collection to draw the co-authorship network depicted in Figure 1. The larger the node of an author, the more publications he or she has in the collection. Thick edges indicate authors that publish intensively together. Accordingly, the co-authorship network gives a fast overview of the author teams found in the specific field of this review.

#### 2.3.1. Articles on psychological intervention programs

Internet-based CBT (iCBT) is an approach that delivers Cognitive Behavioral Therapy via the internet. With 28 articles found, this method currently is the best documented and most researched treatment option when it comes to internet-based services. Of these, 15 articles providing an overview of the main findings are discussed in section 3.

Acceptance and Commitment Therapy (ACT) can be delivered in a self-help format via the internet as well (Westin et al., 2011). One article was found that examined and discussed this in the context of tinnitus treatment.

In another study (Hesser et al., 2012), internet-based ACT (iACT) was compared with iCBT and a moderated online discussion forum. The study revealed substantial improvements for both iCBT and iACT, with no significant difference between the two treatments.

#### 2.3.2. Articles about the Tinnitus E-programme

The Tinnitus E-Programme (TEP) is an internet-based intervention program that was developed in 2009 and evaluated using the Tinnitus Handicap Inventory (THI) (Newman et al., 1996). The program mainly consists of educational contents (i.e., about tinnitus or “the role of psychological mechanisms in tinnitus” (Greenwell et al., 2015), and relaxation- and attentional-focus exercises. The contents are provided as PDF sheets or mp3 audio files. All of the files are accessible for free on a website created for this intervention program [http://www.tinnituseprogramme.org/]. Two articles dealing with TEP were found: in Greenwell et al. (2015) the program is presented, while in Greenwell et al. (2016b) users' reactions and their daily use of relaxation techniques are reported.

#### 2.3.3. Articles on smartphone-delivered sound therapy

With technological advances in the development of smartphones, sound therapy is an easily accessible treatment option. There are smartphone applications that claim to reduce tinnitus loudness by the usage of tailor-made notched music (e.g., Tinnitracks [www.tinnitracks.com] or Tinnitus Pro: Music Therapy [www.promedicalaudio.com]) or other forms of sound therapy (Linnan et al., 2015). Two studies provided the patients with tinnitus masking technologies: Yoo et al. (2015) and Mahboubi et al. (2012b).

Another usage of smartphones related to sound therapy are hearing aids that can be controlled via the smartphone via bluetooth. Further, they include volume-control functions as well as tinnitus maskers. An example of this usage is presented in Sauer et al. (2014). A study that further investigates this technology is provided by Barozzi et al. (2016).

#### 2.3.4. Studies about smartphone-based tinnitus assessment

Diagnosis and assessment of tinnitus are important for a successful treatment. The articles of this category encompass: (1) internet-based hearing tests, namely Mahboubi et al. (2012a), Wunderlich et al. (2015), and Hauptmann et al. (2016), which investigate the potential of internet-based tests in comparison to tests run in the lab; (2) ecological momentary assessments, namely Henry et al. (2012); Pryss et al. (2015a), Schlee et al. (2016); Schickler et al. (2016a), and Wilson et al. (2015); (3) summarizations of the self-measures done by the patients, namely Peter et al. (2016), (4) serious games, namely in Schickler et al. (2016b), and Wise et al. (2015, 2016), (5) a comparison of online vs. paper-based collection of questionnaire data, namely Handscomb et al. (2016) and (6) a study on response times for a web-based emotional Stroop task for tinnitus patients Andersson et al. (2005).

#### 2.3.5. Smartphone apps related to tinnitus

As explained in the last part of section 2.2, three different stores for smartphone applications were searched for tinnitus-related content.

The amount of apps excluded from the initial results vary between the different stores. While there was not much discrepancy between excluded apps from Google's PlayStore (2 of 146 results) and Apple's AppStore (1 of 100 results for iPhone, 4 of 90 results for iPad), Microsoft's Windows 10 Mobile Store displayed a greater number of unrelated applications (58 of 61 results). Most of the irrelevant results in Microsoft's Store included the word “status,” which has the same word ending as “tinnitus.” This leads to the assumption that search was automatically expanded by similar keywords.

Aside from the amount, we investigated all of the apps in greater detail to count the apps that claim to have a tinnitus masker functionality. This analysis revealed that a large number of apps (104 of 144 in the PlayStore, 66 of 99 in the AppStore for iPhones, 48 of 86 in the AppStore for iPad and 3 of 3 in the Windows 10 Mobile Store) included a masker function in their feature set. Other than maskers, many apps were designed to provide information to the user via news articles, videos or photos. Many apps that were included in the results might be appropriate for tinnitus matching, others require specific kinds of hearing aids, functioning as a control unit for said devices. Very few apps included exercises aiming to treat the tinnitus via hypnosis.

## 3. Discussion

As shown in section 2.3, internet-based and smartphone-based technologies are used in intervention programs, whereupon iCBT is the most widespread intervention form, as well as for the recording of assessments, whereupon the recordings are often used for diagnostic purposes (e.g., to match the tinnitus frequency) and for self-help support. As discussed below, the new technologies affect (a) the indicators, symptoms and assessments being collected during an intervention or for diagnostic purposes, and (b) the form and intensity of patient involvement.

### 3.1. Influence of technologies on the amount and form of collected patient information

#### 3.1.1. Assignments and assessments

Similarly to Lui et al. (2017), we found that CBT and its variants like ACT play a central role among the psychotherapeutic treatments for tinnitus. After the publication of the core article of Andersson and Kaldo (2004) on internet-based CBT (iCBT) for tinnitus, there has been a proliferation of studies on enriching CBT with internet-based functionalities: individual weekly treatment plans are considered by Rheker et al. (2015), homework assignments are suggested by Abbott et al. (2009), while patient chats are presented by the aforementioned authors and by Hesser et al. (2012).

Next to assignments, internet technologies and smart devices are used to record patient assessments. Peter et al. (2016) propose PRISM (Pictorial Representation of Illness and Self Measure), a two-dimensional pictorial method for assessing the “burden of suffering” of tinnitus patients. PRISM has been designed for interactive self-assessment with help of an iPad. Whilst the aforementioned iCBT studies put emphasis in the *nature and purpose* of the assignments given to the patients (treatment plans, homework assignments), Peter et al. (2016) also elaborate on the modalities for *patient-app-interaction*, i.e., on the utilities for showing and filling the questionnaire.

Ecological Momentary Assessments (EMAs) for tinnitus patients are investigated by Probst et al. (2016a,b); Schlee et al. (2016) and Wilson et al. (2015) with the goal of understanding tinnitus physiology and moment-to-moment evolution. All these studies used smartphone technology. The EMA analyzed by Probst et al. (2016a,b), and Schlee et al. (2016) are recorded with a dedicated mHealth app, i.e., Track Your Tinnitus. Similarly to the study of (Peter et al., 2016) on PRISM, many studies on EMA under Track Your Tinnitus investigate the patient-app-interaction, aiming at usability and minimal cognitive patient effort (Pryss et al., 2015b; Schlee et al., 2016; Schickler et al., 2016a). Among them, Schickler et al. (2016b) investigate the potential of serious games, while Schickler et al. (2016a) focus on smartwatches, which are less obtrusive than smartphones and can further introduce additional functionality, e.g., monitor vital signs. These studies indicate the importance of enhancing personal experience in the interaction between the tinnitus patient and the mechanism recording assessments or delivering tasks.

#### 3.1.2. Patient recordings for diagnosis and intervention

Hauptmann et al. (2016) present a review of mobile apps that are used for measuring tinnitus pitch. The studies of Mahboubi et al. (2012a) and Yoo et al. (2015) investigate tinnitus masking and consider smart technologies with which the patients themselves can identify the tinnitus frequency they are experiencing. Wunderlich et al. (2015) consider ipod-based tinnitus pitch matching. In the study of Mahboubi et al. (2012a), the patients were asked to match their tinnitus frequency using a web-based protocol. Furthermore, they were subsequently provided with a “customized Harmonic Sound Therapy file” Mahboubi et al. (2012b), the patients listened for 1 h^1^. These studies demonstrate the potential of internet-based and smart technologies for tinnitus matching, but also stress the need for a reliable interaction between patient and technology.

### 3.2. Influence of technologies on the extent and form of patient participation

Internet-based technologies and smartphones allow for a personalized interaction between patient and eHealth/mHealth application. In the literature we investigated, this led to questions about the role of the therapist and the effect of self-help, and about patient involvement and attrition, as described hereafter.

#### 3.2.1. Therapist assistance and self-help

The role of the therapist has been investigated in the context of iCBT by Kaldo et al. (2008), Hesser et al. (2012), and Rheker et al. (2015) among others, while Jasper et al. (2014) juxtaposed self-help to group-based CBT. A remarkable finding is reported by Rheker et al. (2015), who studied the alternatives of “support-on-demand” and “no-support” and found no differences between the two options.

As opposed to iCBT and similar forms of treatment, self-help apps rely entirely on active patient involvement, whereupon the response rate, once and over a long range of time, is used as indicator of success: Wilson et al. (2015) report a response rate of 79.4% for ECA (889 out of 1120 questionnaires were returned), while Pryss et al. (2015b) report that 90% of the questionnaires filled under Track Your Tinnitus come from 18% of the users and thus, incentives for patient involvement need to be established. Advances in that domain include serious games (Schickler et al., 2016b) and passive forms of crowdsensing, as used for other diseases (e.g., diabetes) to monitor vital signals.

#### 3.2.2. Patient participation and attrition

An aspect of substantial interest in the identified literature concerns participation and attrition. Number of participants and percentage of dropouts are used as key indicators of iCBT efficancy: in the studies of Abbott et al. (2009), Hesser et al. (2012), Weise et al. (2012, 2016), Kaldo et al. (2013), Jasper et al. (2014), Beukes et al. (2015, 2016), Rheker et al. (2015), and Heinrich et al. (2016), the number of participants ranges between 44 and 293 (average:113). The drop-outs are depicted in the rightmost column of Table 1.

**Table 1:** Participation and attrition (number of dropouts) in the inspected iCBT studies.

**Literature source**	**#Participants**	**#drop-outs**
Hesser et al., 2012	99	10
Rheker et al., 2015	112	9 in the support-on-demand group (out of 56), 11 in the no-support group (out of 56)
Weise et al., 2012	124	5
Jasper et al., 2014	128	7
Beukes et al., 2016	44 (of which 37 completed the screening questionnaire)	15
Weise et al., 2016	124	5
Heinrich et al., 2016	112	14

As can be seen in the last two columns of Table 1, the variance is substantial. This is in agreement with the findings in the review of Greenwell et al. (2016a, p. 84), where large differences are reported among the surveyed studies as well.

Self-help apps like (Pryss et al., 2015b) and Wilson et al. (2015) do not have an explicit notion of dropout, the patient decides whether or not to use the app. However, patient involvement is not less mission-critical. The observation of Pryss et al. (2015b) that 90% of the Track Your Tinnitus questionnaires come from 18% of the app users indicates a very skewed distribution. However, this does not directly imply patient dissatisfaction; patients would most likely give up the app if they do not need it anymore. Hence, the response rates of self-help apps call for further investigation of the factors modulating attrition.

### 3.3. Technical challenges

#### 3.3.1. Platform diversity

As with many smartphone applications, software for tinnitus monitoring, delivery of information materials, and data collection need to take the platform and application interfaces into account. Compatibility with each smartphone operating system translates into additional costs for software development and maintenance. Interaction with hearing aids, sensors, smartwatches and other devices causes further costs and requires expertise. Furthermore, the user interfaces vary substantially: the design of fill-in forms, especially of those requiring free-text, must be re-considered when input is acquired through smartphones and smartwatches.

#### 3.3.2. Non-obtrusive data acquisition

Smart technology is appropriate for the acquisition of data without active user involvement, e.g., from the microphone, camera, gyro-sensors, and accelerometers. Such data can be used for tinnitus monitoring without the need to increase user participation. However, data protection and anonymization of the data needs to be addressed.

For the monitoring of other chronical diseases, such as diabetes, the use of non-obtrusive data acquisition technologies is widespread as reported by Brzan et al. (2016). Tinnitus monitoring is less widespread, but gains momentum, as our findings show. Moreover, the collection of data about sleep, social interaction, and behavioral patterns are relevant as well, as reported e.g., by Wang et al. (2014) and Lane et al. (2011): the findings from such initiatives are also of relevance for the support of tinnitus patients, since tinnitus has been found to “. [be] engendering the sense of cognitive and emotional reactions” as pointed out by Ghodratitoostani et al. (2016), who also list insomnia among the consequences.

## 4. Summary, limitations and outlook

Our review has shown that internet-based and smart technologies are intensively investigated for the potential to support tinnitus patients. The technologies are used during treatment, e.g., to assign tasks to the patients, but also to collect patient assessments, including Ecological Momentary Assessments. The technologies are also used for diagnostic purposes, e.g., for tinnitus matching or to monitor tinnitus evolution over time.

We found that these new technologies influence the amount of collected patient information but also the form of the interaction. There is a thread of studies on internet-based and smart technologies as part of a physician-supervised tinnitus therapy, and a younger thread on self-help solutions for patients. In both threads, there is awareness about the need for dedicated questionnaire designs (in the context of tinnitus monitoring) and to a more limited extent, awareness about the need to cope with diversity of platforms and user interfaces.

The use of internet-based and smart technologies also affects the extent and form of patient participation. We identified studies that investigate the role of therapist assistance and that juxtapose therapy design with and without therapist. The role of patient participation is naturally a central subject in these studies. There are research contributions that simply report on the number of drop-outs from a conducted field study, whereby the variance is high. But the new technologies are also used outside the scope of typical clinical studies: research papers that report on such technology use are mainly concentrating on self-help eHealth/mHealth apps; they measure response rate of the patients rather than drop-outs, and identify also a high variance.

The co-authorship network of Figure 1 highlights the teams of authors that work intensively on the potential of new technologies for tinnitus. The graph shows that teams build clusters with strong links (thick and intensively colored) among the members, indicating intensive joint publication activity, while there are only few and weak links between clusters. These clusters seems to correspond to the topics being investigated, according to our findings. In particular, the cluster with the strongest links corresponds to the most intensive thread of research; the authors in this cluster work together to investigate the role of internet-based and smart technologies for tinnitus treatment, with emphasis on iCBT. The cluster with the second-strongest links (bottom of the figure) contains authors that work together to understand tinnitus through EMA but at the same time investigate the patient-app-interaction.

A limitation of our review lays in the use of Google Scholar as primary source, whereupon only the top-20 articles were considered. It is possible that relevant articles were overlooked, because they were not in the top-20 positions or because they were not indexed in that query engine. The focus on keyword-based retrieval of scientific works implies that our review did not cover studies on smart technologies for chronical diseases with resemblance to tinnitus nor for comorbidities of tinnitus (like depression), but without explicit reference to the keyword “tinnitus.” Research articles that investigate the potential of dedicated devices are underrepresented: studies that consider dedicated hardware next to smart technology, e.g., for tinnitus masking, are covered in our work, but studies that only consider dedicated hardware are not.

Our market overview is biased by our inclusion criterion, namely the keyword “tinnitus”: we did not consider mobile apps that can be used for tinnitus masking and for relaxation but do not refer explicitly to tinnitus. At the same time, our overview did not address the veracity of the texts we acquired. We took the perspective of the non-expert, potential customer for those apps, and did not verify the claims made in the app description. We strongly believe that certification of such apps is necessary to protect patients from misleading statements, but such a task was beyond the scope of our study.

We plan to extend our work by investigating how smart technologies are used in other chronical diseases that show resemblances to tinnitus. The notion of resemblance has to be specified crisply, but our first focus will be on diseases that require patients to fill-in questionnaires frequently (e.g., diabetes) and diseases for which ambient recordings are collected and exploited in a non-obtrusive way (e.g., dementia).

## Author contributions

SK and MS created the search strategies. SK created the networks-graphics and drafted the initial manuscript. All authors contributed equally to all other stages of the manuscript development, produced, and approved the manuscript.

### Conflict of interest statement

The authors declare that the research was conducted in the absence of any commercial or financial relationships that could be construed as a potential conflict of interest. The handling editor is currently co-organizing a Research Topic with some of the authors WS, RP, TP, MR, BL, and MS, and confirms the absence of any other collaboration.

## Footnotes

^1^Mahboubi et al. (2012a) showed a temporary reduction in tinnitus loudness in 26 of their 32 participants, and in tinnitus annoyance in 27 of 32 participants, leading to the conclusion that internet-based, customized sound therapy can be effective in reducing tinnitus annoyance and loudness.
